# Green synthesis of selenium nanoparticles with extract of hawthorn fruit induced HepG2 cells apoptosis

**DOI:** 10.1080/13880209.2018.1510974

**Published:** 2018-11-02

**Authors:** Dongxiao Cui, Tingting Liang, Liqian Sun, Liqiang Meng, Congcong Yang, Liwei Wang, Taigang Liang, Qingshan Li

**Affiliations:** a School of Pharmaceutical Science, Shanxi Medical University, Taiyuan, PR China;; b Shanxi Provincial Hospital of Traditional Chinese Medicine, Taiyuan, PR China;; c Shanxi University of Chinese medicine, Jinzhong, PR China

**Keywords:** Trace element, antitumor, human liver cancer, oxidative stress, mitochondrial pathway

## Abstract

**Context:** Selenium nanoparticles (SeNPs) have attracted worldwide attention due to their unique properties and potential bioactivities. Considering that hawthorn is both a traditional medicine and a common edible food, hawthorn fruit extract (HE) was chosen as a reductant to prepare SeNPs.

**Objective:** SeNPs were synthesized by using an aqueous HE as a reductant and stabilizer. The antitumor activities and potential mechanisms of SeNPs were explored by using a series of cellular assays.

**Materials and methods:** The HE mediated SeNPs (HE-SeNPs) were examined using various characterisation methods. The cytotoxicity was measured against HepG2 cells after treated with 0, 5, 10 and 20 μg/mL of HE-SeNPs for 24 h. Annexin V-FITC/PI staining analysis was performed to observe the apoptosis of HepG2 cells. Additionally, mitochondrial membrane potential (MMP), intracellular reactive oxygen species (ROS) levels were evaluated. Finally, the protein expression levels of caspase-9 and Bcl-2 were identified by Western blot.

**Results:** The mono-dispersed and stable SeNPs were prepared with an average size of 113 nm. HE-SeNPs showed obvious antitumor activities towards HepG2 cells with an IC_50_ of 19.22 ± 5.3 μg/mL. Results from flow cytometry revealed that both early and total apoptosis rates increased after treating with HE-SeNPs. After cells were treated with various concentrations of HE-SeNPs (5, 10 and 20 μg/mL) for 24 h, the total rate increased to 7.3 ± 0.5, 9.7 ± 1.7 and 19.2 ± 1.6%, respectively. Meanwhile, treatment of HE-SeNPs up-regulated intracellular ROS levels and reduced the MMP. In addition, HE-SeNPs induced the up-regulation of caspase-9 and down-regulation of Bcl-2.

**Discussion and conclusions:** HE-SeNPs induced intracellular oxidative stress and mitochondrial dysfunction to initiate HepG2 cell apoptosis through the mitochondrial pathway. Therefore, HE-SeNPs may be a candidate for further evaluation as a chemotherapeutic agent for human liver cancer.

## Introduction

The field of nanotechnology has seen tremendous growth over the past decade and the recent applications of nanotechnology in medicine have offered exciting possibilities in healthcare (Shi et al. 2010; Wong et al. 2010). Being an essential trace element for the human body, selenium (Se) generally acts as a co-factor and is present in some enzymatic structures called selenoproteins *in vivo* (Beckett and Arthur 2005; Sarkar et al. 2015; Zeliha et al. 2015). In the last two decades, Se has attracted considerable attention due to its clear health benefits (Talas et al. 2009, 2013; Ozdemir et al. 2010), particularly in the area of cancer prevention (Rayman 2000, 2005; Gao et al. 2014). However, there is a very narrow margin between activity and toxicity (Lanctot et al. 2017). Compared with general inorganic Se and organic Se, selenium nanoparticles (SeNPs) display better bio-availability, higher biological activity and lower toxicity (Wang Y et al. 2013; Yu S et al. 2015). Recent studies have shown that nanoselenium indicated potential bio-activities, especially antitumor properties (Yu B et al. 2012; Huang et al. 2013; Pi et al. 2013).

Currently, the main methods for preparing nanoselenium include chemical reduction, sonochemical process (Li et al. 2005) and radiolysis reduction (Zhu et al. 1996). Unfortunately, all of these methods still face many problems, such as hazardous products produced by chemical reagents, ultrasound and radiation. Therefore, it is urgent to develop a green method for the synthesis of SeNPs (Mukherjee et al. 2001). In recent years, the synthesis of nanoparticles using plant extracts and microorganisms has been a possible alternative to chemical and physical methods. Researchers have found that microorganisms can be applied as potential bioreactors for synthesis of metal/metalloid nanoparticles. They have successfully synthesized SeNPs by using *Klebsiella pneumoniae* and *Bacillus cereus* (Dhanjal and Cameotra 2010; Jafari et al. 2010). Similar to microorganisms, plant extracts can also be as an alternative solution for the preparation of nanoparticles (Njagi et al. 2011; Veerasamy et al. 2011). Wang et al. (2017) reported that the functional nanoscale zero-valent iron was satisfactorily fabricated by directly introducing high pure tea polyphenol as reductant. Another study reported the synthesis of silver nanoparticles using the bark extract and powder of cinnamon (*Cinnamomum zeylanicum* Bl. Bijdr.) (Sathishkumar et al. 2009). However, reports on the green synthesis of SeNPs by using plant extracts are relatively limited.

Hawthorn fruit extract (HE) refers to the aqueous red berries extracts of hawthorn (*Crataegus hupehensis* Sarg.); it is nontoxic and edible. It has served as a food and in traditional medicine for a long time. In this article, HE mediated SeNPs (HE-SeNPs) were synthesized *via* a facile, eco-friendly and low-cost approach in which HE acted as the reductant and stabilizer. In addition, the antitumour activity and the underlying antitumour mechanisms of HE-SeNPs against HepG2 cells were further investigated.

## Materials and methods

### Regents and materials

Fetal bovine serum (FBS), Dulbecco’s modified Eagle medium (DMEM), trypsin and MTT kit were obtained from Solarbio Science & Technology Co., Ltd. (Beijing, China). Sodium selenite was purchased from Sigma (St. Louis, MO). Annexin V-FITC/PI apoptosis detection kit, reactive oxygen species (ROS) and mitochondrial membrane potential (MMP) kit assay were purchased from Beyotime Institute of Biotechnology (Shanghai, China). Antibodies against caspase-9 and Bcl-2 were obtained from Cell Signaling Technology (Beverly, MA). Antibody against β-actin, anti-rabbit and anti-mouse secondary antibodies were purchased from Bioworld Technology, Co., Ltd. (Nanjing, China).

### Cell line and cell culture

The HepG2 (human liver carcinoma) and HL02 (normal liver cell) cell line were purchased from the Institute of Biochemistry and Cell Biology, Chinese Academy of Sciences. The HepG2 cells were cultured at 5% CO_2_ and 37 °C in DMEM supplemented with 10% FBS, 100 U/mL penicillin and 100 μg/mL streptomycin.

### Synthesis of HE-SeNPs

For the preparation of HE, dried hawthorn fruits (10 g) were cut into small pieces and soaked overnight with 200 mL ultrapure water. Then, the system was refluxed for 2 h. The obtained extract was filtered three times and then the solution was lyophilized by vacuum freeze dryer for further use. For the synthesis of hawthorn extract mediated SeNPs (HE-SeNPs), 0.01 M sodium selenite was mixed with 2 mg/mL HE under magnetic stirring for 12 h. The obtained solution of HE-SeNPs was dialysed (MWCO 8,000–14,000) in ultrapure water for 48 h to remove the excess sodium selenite. The dialysed HE-SeNPs were characterized by using spectroscopic and microscopic methods. Furthermore, the HE-SeNPs were lyophilized by vacuum freeze dryer (SIM International Group, Miami, FL, USA), and the obtained solid products were utilized in the following experiments.

### Characterisation of HE-SeNPs

Transmission electron microscopy (TEM) samples were prepared by dropping the nanoparticles colloids (10 μL) onto copper grids and dried under infrared light. The photograph was available at an accelerating voltage of 80 kV on JEM-1011 electron microscopy (JEOL, Tokyo, Japan). The size distribution and zeta potential of the SeNPs were examined using the Malvern Zetasizer Nano ZS (Worcestershire, UK). The UV–vis absorption spectrum of the HE-SeNPs was acquired using a scanning spectrophotometer (Mapada, Shanghai Shi, China) with a 1 cm path length. Energy dispersive X-ray spectroscopy (EDS) was carried out to detect the elemental composition of HE-SeNPs. The analysed sample was firstly photographed by SEM and then the selected photograph was subjected to elemental analysis by EDS (Bruker Inc., Madison, WI).

### MTT assays

Anti-proliferation effects of HE-SeNPs were explored with the MTT assay. Cells were cultured in 96-well microplates for 24 h at a density of 1 × 10^5^ cells per well. The HepG2 cells were then incubated with HE and HE-SeNPs at selected concentrations for 24 h. The HL02 cells were incubated with HE-SeNPs at selected concentrations for 24 h. After incubation, 10 μL 5 mg/mL MTT was added and the cells were incubated for further 4 h. Furthermore, 100 μL DMSO was added to dissolve the blue-purple crystal. The optical density (OD) values were measured at 490 and 570 nm using a microplate spectrophotometer (Model 680, Bio-Rad, Hercules, CA).

### Flow cytometric analysis

Cell apoptosis was measured using an Annexin V-FITC/PI apoptosis detection kit (Yin et al. 2015). HepG2 cells (1 × 10^6^) were cultured in a six-well plate and incubated for 24 h. After treating with 0, 5, 10 and 20 μg/mL of HE-SeNPs for 24 h, the cells were incubated with Annexin V and PI for 15 min in dark. Then, the treated cells were observed to determine the apoptosis rate using a Flow Cytometer (Becton Dickinson, Franklin Lakes NJ).

### Intracellular reactive oxygen species (ROS) detection

The intracellular ROS was detected by measuring the conversion from 2′, 7′-dichloro fluorescin diacetate (DCFH-DA) to dichloro fluorescin (DCF) (Zhornik et al. 2015). After indicated treatment, the cells were incubated with 10 μM DCFH-DA for 30 min at 37 °C. Then, the flow cytometry was performed to measure the fluorescence intensity with excitation at 488 nm and emission at 530 nm.

### Mitochondrial membrane potential (MMP) measurement

MMP was evaluated using JC-1 staining method (Zhang et al. 2014). After indicated treatment, the cells were collected and incubated with 500 μL binding buffers containing 5 mg/mL of JC-1 for 20 min at 37 °C. Then, the cells were washed twice to remove the remaining dye, and subjected to analyse by flow cytometry. Finally, the red/green fluorescence ratios were presented to show the MMP levels.

### Protein extractions and Western blot

Expression levels of intracellular apoptosis-related proteins were detected by Western blotting. After the indicated treatment, HepG2 cells were washed with cold PBS and treated with lysis buffer. Then, the cell lysate was centrifuged at 12,000 rpm for 15 min at 4 °C and the total protein was quantified by using the BCA assay. The proteins were separated by 12% SDS-polyacrylamide gel electrophoresis and then transferred to 0.45 μm nitrocellulose membranes. After blocking with 5% nonfat milk for 2 h, the membranes were incubated with TBST containing specific primary antibodies overnight at 4 °C. Then, the membranes were incubated with horseradish peroxide (HRP)-conjugated secondary antibodies, followed by washing three times with TBST buffer. Moreover, enhanced chemiluminescence (ECL) detection kit was performed to examine the target proteins on the X-ray film.

### Statistical analysis

All experiments were repeated at least three times and the results were expressed as mean ± SD. Analysis of variance was carried out using Student’s *t*-test with Origin version 7.0 (Northampton, MA, USA). Differences with *p < 0.05 and **p < 0.01 were considered statistically significant.

## Results and discussion

### Preparation and characterization of nanoparticles

**Figure 1.**
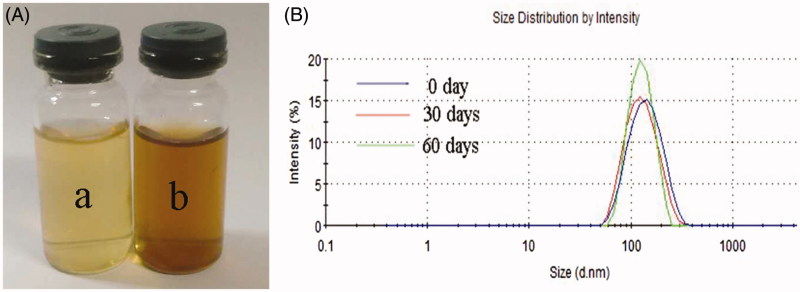
Formation of HE-SeNPs. (A) Photographs of HE (a) and HE-SeNPs (b) aqueous solutions. (B) The particle size distribution measured by laser particle analyser. 0 Day: immediately after sample synthesis.

**Figure 2.**
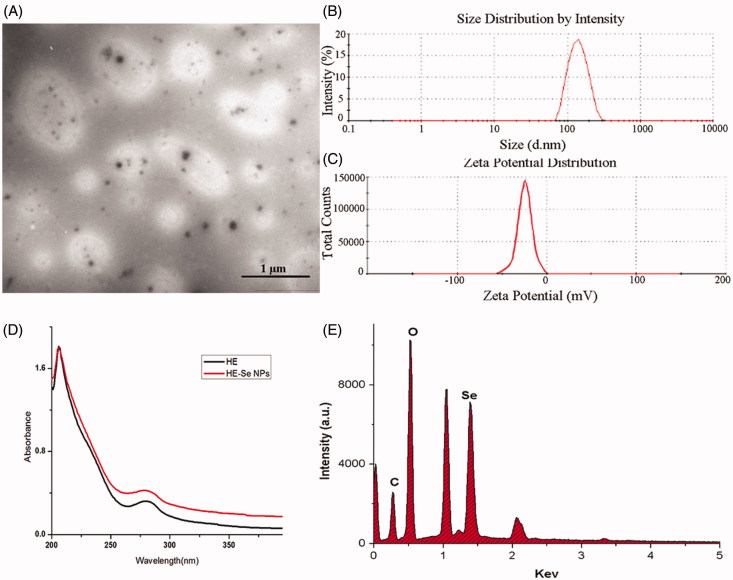
Synthesis and characterization of HE-SeNPs. (A) TEM images of HE-SeNPs, scale bar = 1 μm. (B) Size distribution and (C) Zeta potential of HE-SeNPs in aqueous solution obtained by laser particle analyser. (D) UV–vis spectra of HE-SeNPs. (E) EDS analysis of HE-SeNPs.

After reacting for 12 h, the almost colourless HE solution (Figure 1(A) [a]) turned to brown (Figure 1(A) [b]) indicating the formation of SeNPs (Kong et al. 2014). As depicted in Figure 1(B), the particle size of HE-SeNPs did not increase within 60 d suggested excellent stability of HE-SeNPs. Then, the microstructure and morphology of HE-SeNPs were observed by TEM. As displayed in Figure 2(A), the TEM photographs indicated that the uniform spherical SeNPs were obtained. The size and zeta potential of SeNPs were 113 nm (Figure 2(B)) and −24.5 mV (Figure 2(C)). In Figure 2(D), both HE and HE-SeNPs solutions exhibited absorption at 210 and–280 nm. The intensity of the absorbance was associated with the quantity of nanoparticles (Overschelde et al. 2013). After reaction, the increase in the absorbance of at 280 nm indicated the formation of SeNPs. The result of elemental analysis was presented in Figure 2(E). HE-SeNPs displayed obvious absorption peak of selenium at 1.37 keV. The analysis revealed the proportion of selenium (25.6%) in HE-SeNPs. The other elements were carbon (24.1%), oxygen (35.6%), sodium (11.8%), etc.

### Cytotoxic effects of HE-SeNPs in HepG2 cells

**Figure 3.**
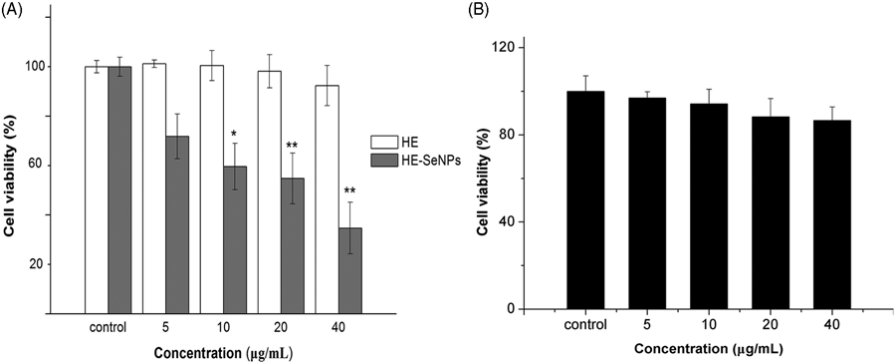
Growth inhibition in HepG2 cells after incubation with HE and HE-SeNPs. (A) The effects of HE and HE-SeNPs on HepG2 cell viability; (B) The effects of HE-SeNPs on HL02 cell viability. The data are presented as mean ± SD (*n* = 3). *p< 0.05 and **p< 0.01 versus the control group.

The growth inhibition of HE-SeNPs in HepG2 cells was evaluated by the MTT assay. As depicted in Figure 3(A), HE had no discernible proliferative inhibition effect in HepG2 cells at the concentrations of 5, 10, 20 and 40 μg/mL. However, HE-SeNPs showed promising antitumor activity towards HepG2 cells with an IC_50_ of 19.22 ± 5.3 μg/mL after 24 h exposures. These results revealed that HE-SeNPs performed significant antitumor activities in HepG2 cells. In addition, HE showed virtually no obvious proliferative inhibition effect in HepG2 cells, which suggested that the antitumor activities of HE-SeNPs were not dependent on HE. It can be presumed that SeNPs played a key role in the anti-tumor activities of HE-SeNPs. Furthermore, the results in Figure 3(B) suggested that He-SeNPs were almost non-cytotoxic to HL02 cells (healthy hepatic cells). To further investigate the anticancer mechanisms of HE-SeNPs against HepG2 cells, the following experiments were carried out.

### HE-SeNPs induced apoptosis of HepG2 cells

**Figure 4.**
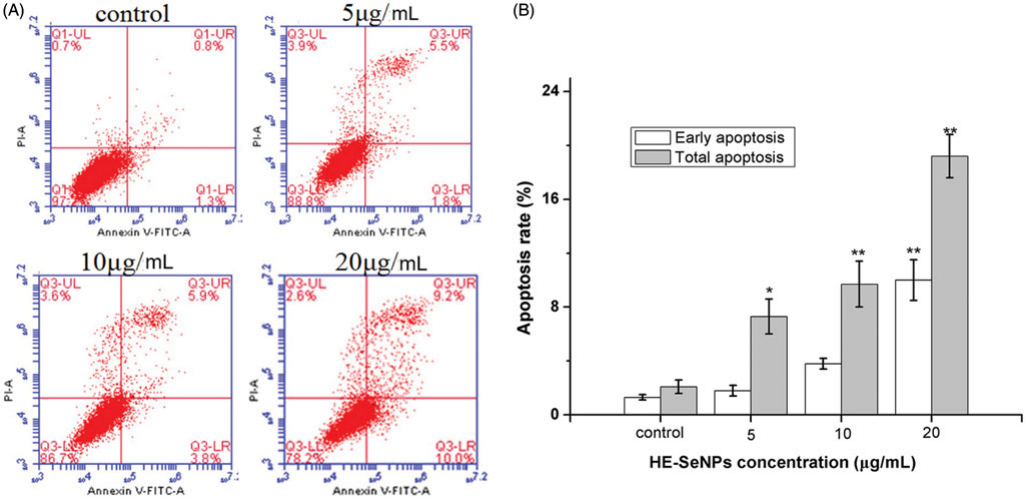
Flow cytometric analysis of HE-SeNPs-induced HepG2 cells apoptosis. (A) Flow cytometry dot plots of Annexin V-FITC/PI double staining for the detection of cell apoptosis. (B) Flow cytometric analysis results. HepG2 cells were incubated with HE-SeNPs at the concentrations of 0, 5, 10 and 20 μg/mL for 24 h. *p < 0.05 and **p< 0.01 versus the control group.

Apoptosis is considered one of the most important mechanisms of the anticancer effect of selenium. To quantitatively determine the apoptosis induced by HE-SeNPs, Annexin V-FITC/PI staining assay was employed to measure the cell apoptosis rates. Apoptosis rate (%) = ([number of apoptotic cells]/[number of total cells]) × 100%. PI is a nucleic acid dye that does not penetrate the intact cell membrane of normal cells or early apoptotic cells but can stain the cell nucleus through the membranes of apoptotic and necrotic cells. Therefore, Annexin V and PI can be applied together to distinguish between early apoptosis (Annexin V positive and PI negative) and late apoptosis (Annexin V/PI double positive). Combined with Figure 4(A,B), proportions of early apoptosis and late apoptosis cells were raised with increased incubation concentrations. Both early apoptosis rates and total apoptosis rates were higher when treated with HE-SeNPs at concentrations of 5, 10 and 20 μg/mL, compared with the control. These results revealed that HE-SeNPs induced significant apoptosis in HepG2 cells. Therefore, HE-SeNPs inhibited HepG2 cells growth mainly through induction of apoptosis.

### HE-SeNPs induced ROS over production and MMP disruption

**Figure 5.**
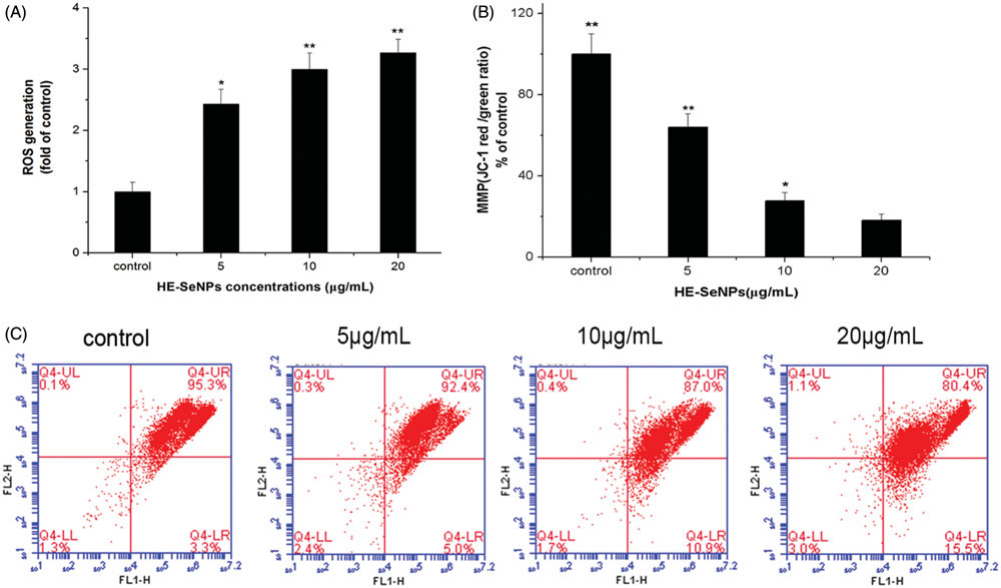
Effects of HE-SeNPs on intracellular ROS and MMP. (A) Representative results for ROS production after treatment. (B) The original flow cytometry results of MMP. (C) The MMP results that were presented graphically as percentages of red/green ratios of the control group. HepG2 cells were incubated with HE-SeNPs at the concentrations of 0, 5, 10 and 20 μg/mL for 24 h. *p < 0.05 and **p < 0.01 versus the control group.

As mentioned above, HE-SeNPs may exert its antitumour activity by inducing apoptosis. In order to elucidate the mechanism of HE-SeNPs-induced apoptosis, the following experiments were implemented. Many current antitumor drugs induce tumour cell apoptosis by increasing ROS levels (Pelicano et al. 2004). Especially, some studies reported that the production of ROS contributed to Se-mediated cytotoxicity (Chen et al. 2008; Wang et al. 2014). Therefore, we measured the ROS levels of HepG2 cells after treatment with HE-SeNPs. As presented in Figure 5(A), treatment of HE-SeNPs up-regulated intracellular ROS level, which suggests the apoptosis induced by HE-SeNPs was related to the generation of intracellular ROS. Over production of ROS may result in mitochondrial dysfunction including MMP loss and activation of the mitochondrial apoptosis pathway.

MMP is critical in maintaining the biological function of cells and the decrease of MMP may trigger the activation of intrinsic apoptosis pathway (You et al. 2010). As shown in Figure 5(B,C), the shift of fluorescence from red to green indicated the MMP rapidly decreased after treatment with HE-SeNPs. This demonstrated that HE-SeNPs triggered apoptosis pathways in HepG2 cells through induction of mitochondria dysfunction. Taken together, these results indicated that HE-SeNPs induced the activation of downstream signalling pathways in HepG2 cells through ROS over production and mitochondrial dysfunction.

### HE-SeNPs induce changes in the expression of apoptosis proteins

**Figure 6.**
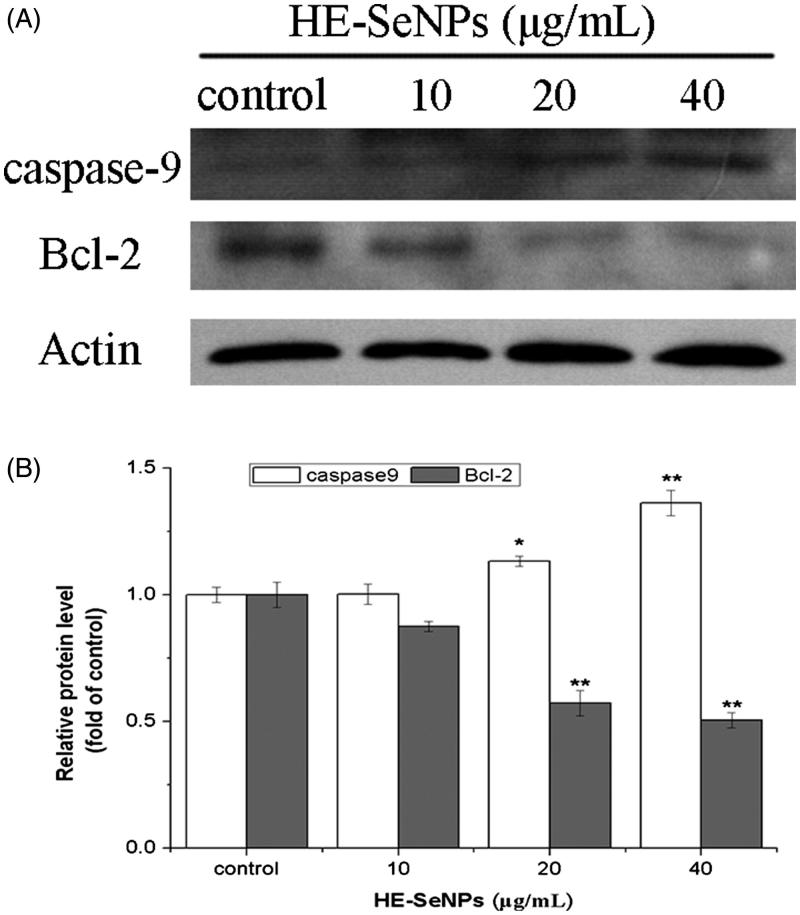
Western blot analysis of caspase-9 and Bcl-2 expression. (A) The immune blots are shown from one experiment representative of three that gave similar results. (B) Densitometry analysis on levels of caspase-9 and Bcl-2 proteins. HepG2 cells were incubated with HE-SeNPs at the concentrations of 0, 10, 20 and 40 μg/mL for 24 h. Each value represents the means ± SD (*n* = 3), *p < 0.05 and **p < 0.01, compared with the control group.

To further demonstrate the apoptosis pathway induced by HE-SeNPs, Western blot was performed to analyse the protein expression of caspase-9 and Bcl-2. As shown in Figure 6, HE-SeNPs induced a dose-dependent increase in caspase-9 levels and a decrease in Bcl-2 levels. Bcl-2 is one of the keys apoptosis proteins involved in mitochondrial pathway, which exerts its bioactivity by inhibiting the release of cytochrome c (Zhen et al. 2016). The decrease of Bcl-2 expression level resulted in increasing mitochondrial membrane permeability and the release of apoptosis activators such as cytochrome c. The increased cytochrome c quickly induces the activation of caspase-9 (Trauzold et al. 2003). Caspase-9 acts as a pro-apoptotic protein, and its activation leads to activation of its downstream caspase-3. The activation of caspase-3 finally results in programmed cell death. Therefore, the increase of caspase-9 expression level and the decrease in that of Bcl-2 indicated the apoptosis was mediated through the intrinsic mitochondrial pathway. In conclusion, these results further prove that HE-SeNPs-induced apoptosis was mediated through mitochondrial pathway with the up-regulation of caspase-9 and down-regulation of Bcl-2.

## Conclusions

In this work, a green method for preparing SeNPs was developed by using HE as the stabilizers and reductant. The obtained HE-SeNPs were mono-disperse spherical morphology and quite stable in aqueous solution with an average diameter of 113 nm. Cytotoxicity assays revealed that HE-SeNPs showed significant antitumor activity against HepG2 cells. Further investigation on antitumor mechanisms demonstrated that HE-SeNPs-induced apoptosis was mediated through the mitochondria pathway with ROS promotion and MMP disruption.
